# Are older long term care residents accurately prognosticated and consequently informed about their prognosis? Results from SHELTER study data in 5 European countries

**DOI:** 10.1371/journal.pone.0200590

**Published:** 2018-07-18

**Authors:** Maud ten Koppel, Bregje D. Onwuteaka-Philipsen, H. Roeline Pasman, Roberto Bernabei, Iain Carpenter, Michael D. Denkinger, Graziano Onder, Henriëtte G. van der Roest, Eva Topinkova, Hein P. J. van Hout

**Affiliations:** 1 Department of Public and Occupational Health, Amsterdam Public Health Research Institute, VU University Medical Center, Amsterdam, The Netherlands; 2 Centro Medicina dell’Invecchiamento, Università Cattolica Sacro Cuore, Rome, Italy; 3 Centre for Health Services Studies, University of Kent, Canterbury, United Kingdom; 4 Agaplesion Bethesda Clinic, Geriatric Centre Ulm/Alb-Donau, Ulm University, Ulm, Germany; 5 Department of General Practice & Elderly Care Medicine, Amsterdam Public Health Research Institute, VU University Medical Center, Amsterdam, The Netherlands; 6 Department of Geriatrics, 1st Faculty of Medicine, Charles University, Prague, Czech Republic; Nord University, NORWAY

## Abstract

**Background:**

Informing residents in long term care facilities (LTCFs) about their prognosis can help them prepare for the end of life. This study aimed to examine which proportion of European LTCF residents, close to death, are accurately prognosticated and consequently informed about their prognosis; and to examine factors related to accurate prognostication and discussion of prognosis.

**Methods:**

A subsample of SHELTER study data was used, consisting of: 500 residents from 5 European countries, who died within 6 months after their last assessment, and had a valid answer on the item ‘End stage disease, 6 or fewer months to live’. This item was used to indicate whether an accurate prognosis was established and discussed with residents. Generalized estimating equations were used to examine factors related to establishment and discussion of accurate prognosis.

**Results:**

86.4% of residents close to death did not receive an accurate prognosis. Residents with cancer; fatigue; dehydration; and normal mode of nutritional intake were more likely to have an accurate prognosis established and discussed. Accurate prognostication and prognosis discussion was less likely for residents who: had a diagnosis under ‘other’; initiated interactions; and residents from Germany, Italy and the Netherlands.

**Conclusions:**

The great majority of residents close to death did not receive an accurate prognosis. Prognostication tools might help clinicians to increase their prognostic accuracy and communication training might help to discuss prognosis with residents.

## Introduction

When approaching the end of life, most older people suffer from chronic diseases which can make their care needs complex and sometimes admission to a long term care facility (LTCF) necessary [1]. Most residents receive care in the LTCF until their death, making end of life (EOL) care and planning for EOL care an important aspect of care in LTCFs [2].

Assessing residents´ life expectancy can help health care professionals to timely start planning EOL care and to provide appropriate care for residents nearing the end of life. Many health organizations state the importance of good communication between patients and healthcare professionals on end of life issues, which includes information about prognosis [3]. Patients prefer to be asked about their preferences for discussing prognosis, before clinicians share this with them [2, 4].

Even though, interviews with older adults revealed that the majority wanted to discuss their prognosis with their clinician. Such information is important for them to prepare for death, make the most of life and to make medical decisions [2].

For persons with chronic diseases, which include LTCF residents, it is often difficult to reliably assess life expectancy because their disease course is not easily predictable near the end of life [5]. Therefore, physicians may be reluctant to establish and discuss a prognosis with patients [6, 7].

A study in French nursing homes showed that 63.5% of residents or their families were informed about the prognosis in the last months before death [8]. It is unknown whether this is comparable in other European countries. Also, the prognostic accuracy is unknown. In addition it is unclear which factors are associated with a lack of establishing and providing an accurate prognosis to LTCF residents.

Therefore this study aimed to: examine which proportion of long term care residents from 5 European countries, who are close to death, are accurately prognosticated and consequently informed about their prognosis; and to examine factors that are related to being accurately prognosticated and consequently informed about prognosis.

## Methods

Research ethics approval for the SHELTER study was received for all participating countries and specifically from the following ethics committees: Medisch Ethische Toetsingscommissie VU Medisch Centrum; Comitato Etico, Universita Cattolica del Sacro Cuore Rome; Tutkimuseettinen Työryhmä, Terveyden Ja Hyvinvoinnin Laitos; Ethics Committee, University of Haifa; Ethikkommission der Universität Ulm; Multicentric Ethics Committee, General Faculty Hospital Prague; Ethics Committee Hospital Saint Périne Paris; School’s Research Ethics Committee, University of Kent Canterbury. Written consent was obtained with assurance of data confidentiality.

### Study setting

The sample for this study was drawn from the Services and Health for Elderly in Long TERmcare (SHELTER) project, which was funded by the Seventh Framework Programme of the European Union [9]. This 12-month prospective cohort study involved seven European countries (Czech Republic, England, Finland, France, Germany, Italy, and the Netherlands) and one non-European country (Israel). In each country a sample of LTCFs willing to participate were identified. Therefore the sample was not randomly selected and not necessarily representative of all LTCFs in these countries. In total 57 LTCFs and 4156 LTCF residents were included.

The study was conducted from 2009 to 2011. Approval for the study was obtained in all participating countries, according to local ethical regulations.

The SHELTER project aimed to validate the interRAI LTCF in a European sample. The interRAI LTCF is a standardized instrument to assess care needs and care provision to LTCF residents. It contains over 350 items, such as: sociodemographic variables; physical, cognitive and psychosocial functioning; clinical diagnoses; treatments; and medication use.

In most facilities data was collected solely as research data and not as routine data. In those facilities data was collected by research nurses independent of the facility. All research nurses were trained for data collection according to the same procedure, which included use of a variety of information sources (personal interviews or observation, chart review and communication with (informal) care givers) to score items.

Older people living in participating LTCFs at the beginning of the study and those admitted in the 3 months enrolment period were assessed by the interRAI LTCF. Residents were then followed-up at 6 months and 12 months. No exclusion criteria were adopted. Residents were invited to participate in the study and were free to decline participation. Written consent was obtained with assurance of data confidentiality.

### Sample

To answer the research questions, a subsample of the SHELTER study sample was selected. This subsample consisted of residents who were close to death: residents who died ≤6 months after their last assessment and who had a valid answer on the interRAI LTCF item ‘End stage disease, 6 or fewer months to live’. Finland, France and Israel were excluded due to selective missing data on the time till death of the last assessment. The study sample comprised 500 residents from Czech Republic, Germany, Italy, the Netherlands and England.

### Outcome measure

The item ‘End stage disease, 6 or fewer months to live’ was used as the outcome variable. This item indicates whether a resident is expected to live ≤ 6 months and also whether this has been communicated with the resident or family. This item was scored with either yes or no. As this study only included residents who actually had died ≤6 months after assessment, scoring ‘no’ was deemed as residents not being accurately prognosticated and consequently informed about prognosis. On the other hand, scoring yes meant that an accurate prognosis was established and provided to residents. Interrater reliability of this item was 0.6 in the SHELTER sample [9].

According to the interRAI LTCF manual, research nurses were instructed the following on scoring this item:

End-stage disease, 6 or fewer months to live: the person or family has been told that in the best clinical judgement of the physician, the person has end-stage disease with approximately 6 or fewer months to live. This judgement should be substantiated by a well-documented disease diagnosis and deteriorating clinical course.

Process: observe the person. Consult staff member, especially the person’s physician. Review any clinical records. Use your clinical judgement to determine whether it is appropriate to ask the person about whether they have an ‘end-stage disease’ [10].

### Independent variables

Independent variables assessed in this research included the following:

Sociodemographic variables: age; gender; and country.

Clinical diagnoses: dementia; other neurological disease; heart diseases; lung disease; infections; cancer; psychiatric disease; other; and number of comorbidities.

Functional status: Cognitive Performance Scale (CPS) (0 intact– 6 very severe impairment) [11]; Communication Scale (0 intact– 8 very severe impairment) [12]; Activities of Daily Living Hierarchy (ADLH) scale (0 no impairment– 6 total dependence) [13]; mode of nutritional intake (normal, impaired (any diet modification was necessary) or artificial (any type of feeding tube or parenteral nutrition was necessary); bladder incontinence (any degree of incontinence, catheter or ostomy present); and bowel incontinence (any degree of incontinence or ostomy device present).

Symptoms: Pain scale (0 no pain– 4 daily excruciating pain) [14]; fatigue (0 none– 4 unable to commence any normal day to day activities); Depression Rating Scale (DRS) (0–14, ≥3, indicating depressive disorders) [15]; dyspnea (0 absent– 3 present at rest); anxiety (present, not present); time asleep during the day (awake all/most of the time—largely asleep/unresponsive); vomiting in last 3 days (present, not present); weight loss (≥5% in last 30 days / ≥10% in last 180 days); dehydration / heightened BUN/Cre ratio; pressure ulcers (none—necrotic eschar).

Psychosocial functioning: pursues involvement in life of facility; initiates interactions with others; positive reaction to interactions initiated by others; adjusts easily to change in routine; average time involved in activities (most, more than two-thirds of the time; some, from one-third to two-thirds of the time; little, less than one-third of time; to none); feels lonely; experienced major life stressors in last 90 days; family or close friends reported feeling overwhelmed by resident’s illness; consistent positive outlook; finds meaning in day-to-day life; and strong and supportive relationship with family.

### Analysis

Frequencies and descriptives were used to describe the study sample and to indicate how many residents were accurately prognosticated and consequently informed about their prognosis.

Generalized estimating equations (GEE) were used to make a logistic regression model, in order to examine factors contributing to residents not being accurately prognosticated and informed about their prognosis. The item ‘End stage disease, 6 or fewer months to live’ was used as the dependent variable. GEE were used in order to adjust for the potential confounding effect of facility, as the data have a nested structure. Country was considered a factor that possibly contributed to the prediction model and was therefore treated as an independent variable in analyses. All GEE analyses were adjusted for the potential confounding effect of timing of the last assessment before death. Model specifications included an exchangeable correlation matrix and a robust covariance matrix estimator.

A forward selection strategy was used to make a prediction model. First univariate analyses were conducted. Because of the large amount of variables, only variables with a p-value <0.1 in univariate analysis were considered for the forward selection procedure. In the prediction model, p-values <0.05 were considered statistically significant. A minimum of 5 cases per parameter added to the prediction model was used as a rule of thumb to estimate the maximum amount of parameters in the model [16].

Continuous or scale variables that did not show a linear relation with ‘End stage disease’, were dichotomized. Odds ratios (OR) and 95% confidence intervals were derived from each analyses.

All analyses were conducted using IBM SPSS Statistics 22 for Windows (IBM SPSS Statistics, IBM Corporation, Chicago, IL).

## Results

The mean age of residents on their last assessment was 84.7 (SD 8.8) years and 352 residents (70.4%) were women. The 500 residents were from Czech Republic (n = 142), Germany (n = 98), England (n = 150), Italy (n = 33), and the Netherlands (n = 77).

### Proportion of LTCF residents that were accurately prognosticated and consequently informed about prognosis

**Fig 1:**
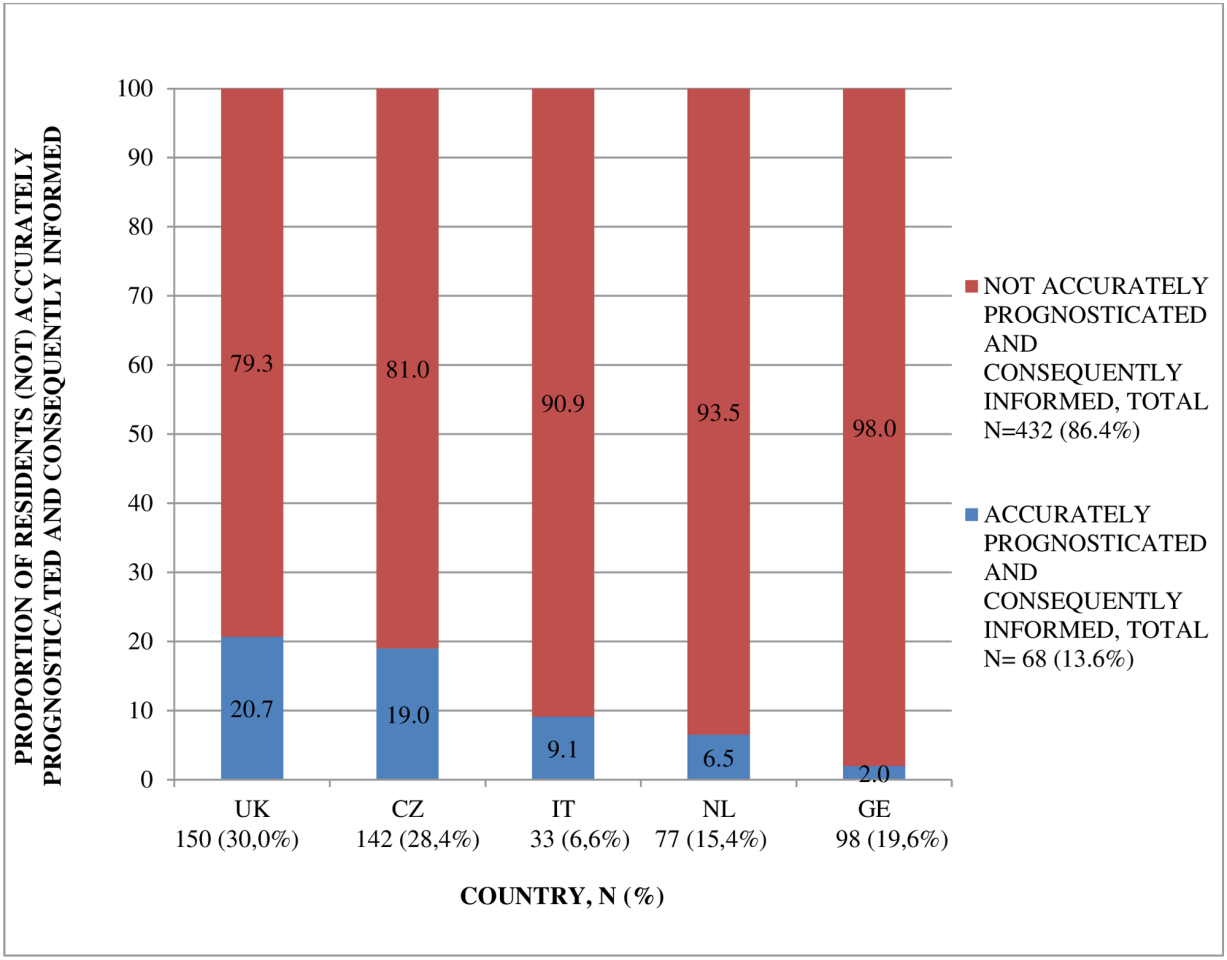
Proportion of residents (not) accurately prognosticated and consequently informed about their prognosis, per country.

**Fig 2:**
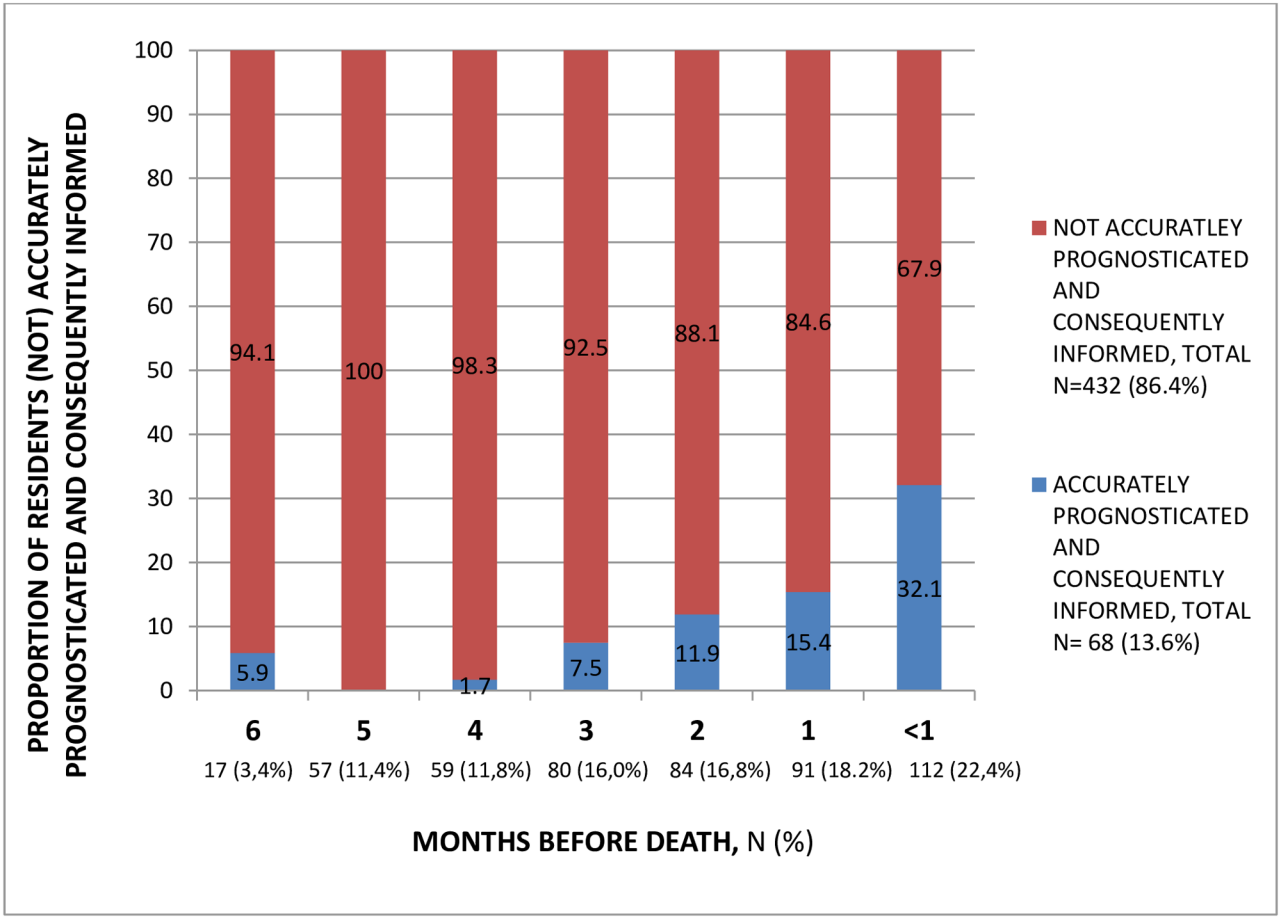
Proportion of residents (not) accurately prognosticated and consequently informed about their prognosis, according to months before death.

Overall, 86.4% of residents were not accurately prognosticated and informed about their prognosis (Fig 1). This proportion was highest in Germany (98.0%) and lowest in England (79.3%). Generally as residents were closer to death, an accurate prognosis was more often established and provided (Fig 2). Of residents with an assessment less than a month before death 32.1% were accurately prognosticated and informed, which was significantly more often compared to residents one to six months before death (p<0.01).

### Factors related to LTCF residents not being accurately prognosticated and consequently informed about prognosis

Univariate analysis showed 19 variables with a p-value <0.1 (see Tables 1–3). Residents with: infections; cancer; cognitive, communication or ADL impairment; artificial or impaired nutritional intake; fatigue; weight loss; dehydration; overwhelmed friends or family; or major life stressors were more likely to be accurately prognosticated and consequently informed about their prognosis.

**Table 1:** Associations between sociodemographic characteristics, diagnoses and aspects of functional status and residents being accurately prognosticated and consequently informed, in univariate analyses.

		Resident accurately prognosticated and consequently informed				Univariate OR (95% CI)^a^	P-value
		Yes n = 68 (13.6%)		No n = 432 (86.4%)			
**Sociodemographic characteristics**							
Gender	Male	20	13.5%	128	86.5%		
	Female	48	13.6%	304	86.4%	1.24 (0.60–2.55)	.558
Age	≤85	40	16.3%	205	83.7%		
	>85	28	11.0%	227	89.0%	0.77 (0.48–1.23)	.274
Country	UK	31	20.7%	119	79.3%		
	NL	5	6.5%	72	93.5%	0.16 (0.05–0.58)	.005
	IT	3	9.1%	30	90.9%	0.59 (0.14–2.40)	.456
	CZ	27	19.0%	115	81.0%	0.39 (0.13–1.13)	.083
	GE	2	2.0%	96	98.0%	0.08 (0.02–0.25)	<.001
**Diagnosis present**							
Dementia	No	38	15.0%	215	85.0%		
	Yes	30	12.1%	217	87.9%	0.90 (0.50–1.61)	.714
Other neurological disease	No	43	13.5%	275	86.5%		
	Yes	25	13.7%	157	86.3%	1.00 (0.52–1.75)	.998
Heart disease	No	41	15.2%	228	84.8%		
	Yes	27	11.7%	204	88.3%	0.69 (0.33–1.43)	.313
Lung disease	No	58	13.2%	383	86.8%		
	Yes	10	16.9%	49	83.1%	1.13 (0.57–2.26)	.725
Infections	No	52	11.8%	389	88.2%		
	Yes	16	27.1%	43	72.9%	1.61 (0.79–3.25)	.188
Cancer^b^	No	34	8.4%	369	91.6%		
	Yes	34	35.4%	62	64.6%	5.22 (2.71–10.06)	<.001
Psychiatric disease	No	51	13.1%	339	86.9%		
	Yes	17	15.5%	93	84.5%	1.32 (0.64–2.73)	.451
Other diagnosis	No	53	14.8%	53	14.8%		
	Yes	15	10.6%	127	89.4%	0.50 (0.28–0.89)	.019
No. of comorbidities	≤4	36	12.5%	253	87.5%		
	(>4)	32	15.2%	179	84.8%	1.07 (0.66–1.75)	.787
**Aspects of functional status**							
Cognitive function	Intact—mild impairment	21	10.4%	181	89.6%		
	Moderate-severe impairment	47	15.8%	251	84.2%	1.77 (1.05–2.99)	.033
Communication	Intact-mild impairment	23	10.4%	199	89.6%		
	Moderate-very severe impairment	45	16.2%	233	83.8%	1.75 (1.15–2.67)	.009
ADL	Independent-limited impairment	5	5.6%	85	94.4%		
	Extensive assistance- total dependence	63	15.4%	347	84.6%	2.29 (1.13–4.64)	.022
Nutritional intake^b^	Normal	16	6.8%	221	93.2%		
	Impaired	42	18.1%	190	81.9%	2.10 (1.15–3.84)	.015
	Artificial feeding	9	30.0%	21	70.0%	2.84 (1.11–7.29)	.030
Bladder incontinence	No	7	11.1%	56	88.9%		
	Yes	61	14.0%	376	86.0%	1.08 (0.50–2.32)	.843
Bowel incontinence^b^	No	12	9.0%	121	91.0%		
	Yes	52	14.4%	310	85.6%	1.52 (.81–2.83)	.192

^a^Logistic regression analyses using Generalised Estimating Equations. Dependent variable: 0 –not correctly classified as having 6 months or less to live, 1 –correctly classified as having 6 months or less to live. Adjusted for time till death.

^b^No. of missing values: Cancer, Nutritional intake: 1; Bowel incontinence: 5

**Table 2:** Associations between residents’ symptom status and residents being accurately prognosticated and consequently informed, in univariate analyses.

		Resident accurately prognosticated and consequently informed				Univariate OR (95% CI)^a^	P-value
		Yes n = 68 (13.6%)		No n = 432 (86.4%)			
Pain	No	30	10.6%	253	89.4%		
	Yes	38	17.5%	179	82.5%	1.59 (0.75–3.36)	.225
Fatigue	No	18	6.5%	260	93.5%		
	Yes	50	22.5%	172	77.5%	3.05 (1.89–4.92)	<.001
Depression	No	43	12.3%	307	87.7%		
	Yes	25	16.7%	125	83.3%	1.51 (0.88–2.59)	.135
Dyspnoea	No	49	12.2%	352	87.8%		
	Yes	19	19.2%	80	80.8%	1.42 (0.87–2.31)	.164
Anxiety	No	60	13.1%	398	86.9%		
	Yes	8	19.0%	34	81.0%	1.55 (0.60–3.97)	.366
Time sleep during the day	Awake all/most time	14	8.4%	153	91.6%		
	Multiple naps—largely asleep	54	16.2%	279	83.8%	1.47 (0.86–2.52)	.162
Vomiting	No	61	13.0%	410	87.0%		
	Yes	7	24.1%	22	75.9%	2.01 (0.82–4.90)	.127
Weight loss	No	38	9.5%	360	90.5%		
	Yes	30	29.4%	72	70.6%	2.72 (1.53–4.84)	.001
Pressure ulcer	No	45	11.1%	360	88.9%		
	Yes	23	24.2%	72	75.8%	1.61 (0.90–2.91)	.110
Dehydrated^b^	No	51	10.9%	419	89.1%		
	Yes	17	58.6%	12	41.4%	9.97 (3.60–27.64)	<.001

^a^Logistic regression analyses using Generalised Estimating Equations. Dependent variable: 0 –not correctly classified as having 6 months or less to live, 1 –correctly classified as having 6 months or less to live. Adjusted for time till death.

^b^No. of missing values: Dehydrated: 1.

**Table 3:** Associations between psychosocial characteristics of residents and residents being accurately prognosticated and consequently informed, in univariate analyses.

		Resident accurately prognosticated and consequently informed				Univariate OR (95% CI)^a^	P-value
		Yes n = 68 (13.6%)		No n = 432 (86.4%)			
Pursues involvement^b^	No	50	17.4%	237	82.6%		
	Yes	15	7.2%	192	92.8%	0.40 (0.23–0.71)	.002
Initiates interactions^b^	No	51	16.8%	252	83.2%		
	Yes	14	7.3%	177	92.7%	0.38 (0.21–0.67)	.001
Reacts positively to interactions^b^	No	29	18.2%	130	81.8%		
	Yes	36	10.7%	299	89.3%	0.60 (0.34–1.06)	.078
Adjusts easily to change in routine^b^	No	40	16.5%	202	83.5%		
	Yes	25	9.9%	227	90.1%	0.63 (0.39–1.02)	.061
Time involved in activities (some—most involvement)^b^	No	55	18.0%	250	82.0%		
	Yes	13	6.7%	181	93.3%	0.52 (0.28–0.97)	.041
Family overwhelmed^b^	No	45	10.8%	373	89.2%		
	Yes	20	26.3%	56	73.7%	2.54 (1.20–5.35)	.014
Lonely^b^	No	47	11.2%	372	88.8%		
	Yes	18	24.0%	57	76.0%	1.99 (0.75–5.25)	.165
Major life stress^b^	No	42	10.4%	360	89.6%		
	Yes	23	25.0%	69	75.0%	1.97 (0.95–4.10)	.069
Consistent positive outlook^b^	No	48	16.1%	250	83.9%		
	Yes	17	8.7%	179	91.3%	0.56 (0.33–0.97)	.037
Finds meaning^b^	No	50	18.7%	217	81.3%		
	Yes	15	6.6%	212	93.4%	0.32 (0.17–0.59)	<.001
Strong relationship with family^b^	No	23	13.9%	142	86.1%		
	Yes	42	12.8%	287	87.2%	0.79 (0.44–1.41)	.417

^a^Logistic regression analyses using Generalised Estimating Equations. Dependent variable: 0 –not correctly classified as having 6 months or less to live, 1 –correctly classified as having 6 months or less to live. Adjusted for time till death.

^b^No. of missing values: Pursues involvement, initiates interactions, reacts positively to interactions, adjusts easily to change in routine, family overwhelmed, lonely, major life stress, consistent positive outlook, finds meaning, strong relationship with family: 6; Time involved in activities: 1

On the other hand, residents who: had a clinical diagnosis categorized under ‘other’; pursued involvement; spent time involved in activities; initiated or reacted positively to interactions; adjusted easily to changes; had a consistent positive outlook on life; found meaning in life; or lived in the Netherlands, Germany or Czech Republic, were less likely to have an accurate prognosis established and shared with them.

The multivariate model (see Table 4) showed that residents with cancer, fatigue, dehydration, or an impaired or artificial mode of nutritional intake, were more likely to be accurately prognosticated and informed about their prognosis. However, the establishment and provision of an accurate prognosis was less likely for residents from Germany, the Netherlands or Italy and residents who had a diagnosis under ‘other’ or initiated interactions.

**Table 4:** Factors related to residents not being accurately prognosticated and consequently informed, in multivariate analyses.

		Multivariate OR (95% CI)^a^	P-value
Country	UK		
	NL	0.25 (0.11–0.58)	.001
	IT	0.18 (0.05–0.66)	.009
	CZ	0.47 (0.17–1.28)	.138
	GE	0.06 (0.01–0.34)	.001
Cancer	No		<.001
	Yes	11.04 (5.34–22.83)	
Mode of nutritional intake^b^	Normal		
	Impaired	2.02 (0.94–4.33)	.073
	Artificial feeding	6.80 (2.17–21.36)	.001
Fatigue	No		.002
	Yes	2.73 (1.45–5.14)	
Dehydrated	No		<.001
	Yes	8.16 (2.52–26.48)	
Diagnosis other	No		.024
	Yes	0.52 (0.29–0.92)	
Initiates interactions	No		.022
	Yes	0.44 (0.22–0.89)	

^a^ Multivariate logistic regression analyses using Generalised Estimating Equations. A forward selection approach was used, entering only variables with p <0.1 in univariate analyses and using p<0.05 as a cut-off point in the multivariate model. N = 492. Dependent variable: 0 –not correctly classified as having 6 months or less to live, 1 –correctly classified as having 6 months or less to live. Adjusted for time till death.

## Discussion

First, results from this study showed that the great majority, namely 86.4%, of LTCF residents, seem not to be accurately prognosticated and consequently informed about their prognosis within 6 months of actual death. Second, residents with: cancer, fatigue, dehydration, or impaired of artificial mode of nutritional intake were more likely to be accurately prognosticated and informed. While residents who initiated interactions, had a diagnosis under ‘other’ or lived in a LTCF in Germany, the Netherlands or Italy were less likely accurately prognosticated and informed.

### Proportion of LTCF residents that were accurately prognosticated and consequently informed about prognosis

Compared to the study conducted in French LTCFs, where 63.5% of residents or families were informed about the prognosis, the proportion of informed residents in this study seems quite low. A study in patients in general practice also showed higher proportions of life expectancy discussions: 23% and 68% of patients in Italy and the Netherlands, respectively [17].

A possible explanation for these differences could be that aforementioned studies only measured whether patients were informed about their prognosis. While the current study also questioned the accuracy of a more specific prognosis, namely ≤6 months to live. Thus scoring negatively on this item could entail several scenarios: a resident was neither prognosticated, nor informed about a prognosis; a resident was prognosticated, but not informed about this prognosis; or a resident had been informed about prognosis in more general terms, instead of specifying to ≤6 months to live. Clinicians have indicated to deal with prognostic uncertainty and rather not use too definitive or descriptive time frames [18]. Furthermore health professionals can feel uncomfortable to discuss prognosis with patients and be afraid it will have negative consequences for the patients [18]. Thus combining prognostication and discussing prognosis, both considered difficult, could have led to the low proportion found in this study.

Furthermore, in the current study the discussion of prognosis was preferably recorded prior to residents’ deaths, while the other studies used retrospective surveys after patients had died. As discussing prognosis with patients is considered desirable [3], not having had such discussions with patients who died, could imply one has not delivered optimal care. Possibly retrospectively filling in whether prognosis was discussed leads to a higher degree of socially desirable answers and thus an overestimation. In the current study residents with an assessment <1 month before death, were significantly more often prognosticated and informed about their prognosis than residents with an assessment 1–6 months before death. Research in nursing home residents with dementia has shown that as residents got closer to death, there was a significant increase of setting palliative care goals [19]. However, <1 month before death still only less than 1/3 of residents received an accurate prognosis, indicating that physicians in the LTCF setting probably have trouble establishing a prognosis, even when residents are nearing death. Possibly residents in the current study were prognosticated and informed about their prognosis in the period between assessment and actual death. Therefore the current study could underestimate actual practice.

As establishing a prognosis is important for clinicians to provide appropriate care and sharing this prognosis is important for patients, recommendations for practice should include both prognostication and communication with patients. Several tools have been developed which can aid clinicians in estimating life expectancy of LTCF residents [20–24]. Communication training has shown to improve clinicians communication skills [25] and could perhaps improve discussing prognosis with residents. A starting point for this training could be the following key elements of discussing prognosis: establish what the patient and family already know about their prognosis; determine whether the patient is ready to discuss prognosis and what the patients wants to know; deliver information clearly; and respond appropriately to a patient’s emotion [26].

### Factors related to LTCF residents not being accurately prognosticated and consequently informed about prognosis

Residents who had cancer were more often prognosticated and informed about their prognosis, while this was less often for residents with a diagnosis under ‘other’. This is concurrent with literature, as cancer patients usually have a more predictable illness trajectory and are easier to prognosticate, they tend to receive more end-of-life information [5, 8, 17, 27].

Furthermore, studies have shown that fatigue, dehydration and artificial or impaired mode of nutritional intake are predictive of mortality in LTCF residents [21, 24, 28, 29]. Thus according to literature, a resident presenting with these factors would indeed represent a resident who is closer to death. This could explain why these residents were more often accurately prognosticated and informed about their life expectancy in the current study.

On the other hand, prognosis was less often accurately established and discussed with residents who still initiated interactions. Possibly clinicians think that these residents are so much engaged in life that they are not ready yet to discuss end-of-life matters and clinicians fear it will have negative consequences. Navigating patient’s readiness and fear of causing distress are known barriers for clinicians in end-of-life communication [30, 31]. However, patients can still enjoy a good quality of life and acknowledge their prognosis [32].

Country of residence was also related to whether or not physicians would establish and discuss prognosis with residents. Differences between countries were also seen in the intention to discuss prognosis with patients [33] and in GPs discussing prognosis [17]. As in general practice, possibly country specific differences in health service organisation and the importance of autonomy could play a role in these differences found between countries [17].

### Strengths and limitations

This is the first study on establishing and discussing prognosis in LTCFs, that used data from several European countries. To our knowledge it was also the first prospective study on this subject. The extensive SHELTER database made it possible to consider an array of factors that might be related to a lack of establishing and discussing prognosis.

Another strength of this study is using forward selection to build the model. The number of parameters that could be entered in a regression model was limited, since only 68 residents scored ‘yes’ on the outcome variable. Backward selection would have required a rigorous preselection of the many variables that were considered, making the analysis less transparent.

A limitation of the item ‘End stage disease, 6 or fewer months to live’, is the impossibility to distinguish between residents who were: prognosticated but not informed on their prognosis; informed about their prognosis but not specifically using a 6-month time window; and not prognosticated and informed about prognosis at all. All these residents would not be scored affirmatively on this interRAI item, but for different reasons the current study cannot differentiate between.

Another limitation that should be mentioned, is the relatively small number of residents from Italy, compared to the samples from the other countries. Cross-country comparisons with Italy and implications from the Italian data, should therefore be interpreted with caution.

### Conclusion

The great majority of LTCF residents who were close to death, were not accurately prognosticated and consequently informed about their prognosis in the current study. While residents closer to death were more often informed about their life expectancy, most residents assessed <1 month before death still did not receive an accurate prognosis. Prognostication tools might help clinicians to increase their prognostic accuracy and communication training might help to discuss prognosis with residents. Future studies might investigate whether residents are perhaps being informed about their prognosis in more general terms early in the disease trajectory and whether prognosis is being updated as a resident’s condition deteriorates. As it is currently not clear whether most difficulties lie with prognostication or with discussing this with residents, future research could distinguish between these matters.

## Supporting information

S1 Dataset (SAV)

## References

[1] Hall S, Kolliakou A, Petkova H, Froggatt K, Higginson IJ. Interventions for improving palliative care for older people living in nursing care homes. Cochrane Database Syst Rev. 2011(3):CD007132 10.1002/14651858.CD007132.pub2 21412898 PMC6494579

[2] Ahalt C, Walter LC, Yourman L, Eng C, Perez-Stable EJ, Smith AK. "Knowing is better": preferences of diverse older adults for discussing prognosis. J Gen Intern Med. 2012;27(5):568–75. 10.1007/s11606-011-1933-0 22127798 PMC3326105

[3] Barazzetti G, Borreani C, Miccinesi G, Toscani F. What "best practice" could be in Palliative Care: an analysis of statements on practice and ethics expressed by the main Health Organizations. BMC Palliat Care. 2010;9:1 10.1186/1472-684X-9-1 20205778 PMC2823604

[4] Clayton JM, Butow PN, Tattersall MH. When and how to initiate discussion about prognosis and end-of-life issues with terminally ill patients. J Pain Symptom Manage. 2005;30(2):132–44. 10.1016/j.jpainsymman.2005.02.014 16125028

[5] Murray SA, Kendall M, Boyd K, Sheikh A. Illness trajectories and palliative care. BMJ. 2005;330(7498):1007–11. 10.1136/bmj.330.7498.1007 15860828 PMC557152

[6] Barnes S, Gott M, Payne S, Seamark D, Parker C, Gariballa S, et al Communication in heart failure: perspectives from older people and primary care professionals. Health Soc Care Community. 2006;14(6):482–90. 10.1111/j.1365-2524.2006.00636.x 17059490

[7] Gott M, Gardiner C, Small N, Payne S, Seamark D, Barnes S, et al Barriers to advance care planning in chronic obstructive pulmonary disease. Palliat Med. 2009;23(7):642–8. 10.1177/0269216309106790 19648222

[8] Morin L, Johnell K, Van den Block L, Aubry R. Discussing end-of-life issues in nursing homes: a nationwide study in France. Age Ageing. 2016;45(3):395–402. 10.1093/ageing/afw046 27013503

[9] Onder G, Carpenter I, Finne-Soveri H, Gindin J, Frijters D, Henrard JC, et al Assessment of nursing home residents in Europe: the Services and Health for Elderly in Long TERm care (SHELTER) study. BMC Health Serv Res. 2012;12:5 10.1186/1472-6963-12-5 22230771 PMC3286368

[10] Morris JN P B-T, Fries BE, Hawes C, Murphy K, Mor V, Nonemaker S, Phillips CD. InterRAI Long-Term Care Facilities (LTCF) Assessment Form and User’s Manual (Version 9.1). Washington, DC: interRAI Publications; 2010 118 p.

[11] Morris JN, Fries BE, Mehr DR, Hawes C, Phillips C, Mor V, et al MDS Cognitive Performance Scale. J Gerontol. 1994;49(4):M174–M82. 8014392 10.1093/geronj/49.4.m174

[12] Frederiksen K, Tariot P, De JE. Minimum Data Set Plus (MDS+) scores compared with scores from five rating scales. J Am Geriatr Soc. 1996;44(3):305–9. 8600202 10.1111/j.1532-5415.1996.tb00920.x

[13] Morris JN, Fries BE, Morris SA. Scaling ADLs within the MDS. J Gerontol A Biol Sci Med Sci. 1999;54(11):M546–M53. 10619316 10.1093/gerona/54.11.m546

[14] Fries BE, Simon SE, Morris JN, Flodstrom C, Bookstein FL. Pain in U.S. nursing homes: validating a pain scale for the minimum data set. Gerontologist. 2001;41(2):173–9. 11327482 10.1093/geront/41.2.173

[15] Burrows AB, Morris JN, Simon SE, Hirdes JP, Phillips C. Development of a minimum data set-based depression rating scale for use in nursing homes. Age Ageing. 2000;29(2):165–72. 10791452 10.1093/ageing/29.2.165

[16] Bentler PM, Chou CP. Practical Issues in Structural Modeling. Sociological Methods & Research. 1987;16:78–117.

[17] Evans N, Costantini M, Pasman HR, Van den Block L, Donker GA, Miccinesi G, et al End-of-life communication: a retrospective survey of representative general practitioner networks in four countries. J Pain Symptom Manage. 2014;47(3):604–19. 10.1016/j.jpainsymman.2013.04.008 23932176

[18] Hancock K, Clayton JM, Parker SM, Wal der S, Butow PN, Carrick S, et al Truth-telling in discussing prognosis in advanced life-limiting illnesses: a systematic review. Palliat Med. 2007;21(6):507–17. 10.1177/0269216307080823 17846091

[19] Hendriks SA, Smalbrugge M, Hertogh CM, van der Steen JT. Changes in Care Goals and Treatment Orders Around the Occurrence of Health Problems and Hospital Transfers in Dementia: A Prospective Study. J Am Geriatr Soc. 2016. 10.1111/jgs.14667 27869300

[20] Hjaltadottir I, Hallberg IR, Ekwall AK, Nyberg P. Predicting mortality of residents at admission to nursing home: a longitudinal cohort study. BMC Health Serv Res. 2011;11:86 10.1186/1472-6963-11-86 21507213 PMC3112069

[21] Flacker JM, Kiely DK. Mortality-related factors and 1-year survival in nursing home residents. J Am Geriatr Soc. 2003;51(2):213–21. 12558718 10.1046/j.1532-5415.2003.51060.x

[22] van Dijk PT, Mehr DR, Ooms ME, Madsen R, Petroski G, Frijters DH, et al Comorbidity and 1-year mortality risks in nursing home residents. J Am Geriatr Soc. 2005;53(4):660–5. 10.1111/j.1532-5415.2005.53216.x 15817014

[23] Levy C, Kheirbek R, Alemi F, Wojtusiak J, Sutton B, Williams AR, et al Predictors of six-month mortality among nursing home residents: diagnoses may be more predictive than functional disability. J Palliat Med. 2015;18(2):100–6. 10.1089/jpm.2014.0130 25380219

[24] Porock D, Parker-Oliver D, Petroski GF, Rantz M. The MDS Mortality Risk Index: The evolution of a method for predicting 6-month mortality in nursing home residents. BMC Res Notes. 2010;3:200 10.1186/1756-0500-3-200 20637076 PMC2913927

[25] Rao JK, Anderson LA, Inui TS, Frankel RM. Communication interventions make a difference in conversations between physicians and patients: a systematic review of the evidence. Med Care. 2007;45(4):340–9. 10.1097/01.mlr.0000254516.04961.d5 17496718

[26] Bernacki RE, Block SD. Communication about serious illness care goals: a review and synthesis of best practices. JAMA Intern Med. 2014;174(12):1994–2003. 10.1001/jamainternmed.2014.5271 25330167

[27] Barnes S, Gardiner C, Gott M, Payne S, Chady B, Small N, et al Enhancing patient-professional communication about end-of-life issues in life-limiting conditions: a critical review of the literature. J Pain Symptom Manage. 2012;44(6):866–79. 10.1016/j.jpainsymman.2011.11.009 22819438

[28] K. T. Prognostic Indicator Guidance (PIG). 2011 10/2011. Report No.

[29] Thomas JM, Cooney LM Jr., Fried TR. Systematic review: Health-related characteristics of elderly hospitalized adults and nursing home residents associated with short-term mortality. J Am Geriatr Soc. 2013;61(6):902–11. 10.1111/jgs.12273 23692412 PMC4059538

[30] Slort W, Schweitzer BP, Blankenstein AH, Abarshi EA, Riphagen II, Echteld MA, et al Perceived barriers and facilitators for general practitioner-patient communication in palliative care: a systematic review. Palliat Med. 2011;25(6):613–29. 10.1177/0269216310395987 21273221

[31] Brighton LJ, Bristowe K. Communication in palliative care: talking about the end of life, before the end of life. Postgrad Med J. 2016;92(1090):466–70. 10.1136/postgradmedj-2015-133368 27153866

[32] Clayton JM, Hancock K, Parker S, Butow PN, Walder S, Carrick S, et al Sustaining hope when communicating with terminally ill patients and their families: a systematic review. Psychooncology. 2008;17(7):641–59. 10.1002/pon.1288 18022831

[33] Voorhees J, Rietjens J, Onwuteaka-Philipsen B, Deliens L, Cartwright C, Faisst K, et al Discussing prognosis with terminally ill cancer patients and relatives: a survey of physicians’ intentions in seven countries. Patient Educ Couns. 2009;77(3):430–6. 10.1016/j.pec.2009.09.013 19850436

